# Mechanism of rift flank uplift and escarpment formation evidenced by Western Ghats, India

**DOI:** 10.1038/s41598-019-46564-3

**Published:** 2019-07-19

**Authors:** Radhakrishna T., Asanulla R. Mohamed, Venkateshwarlu M., Soumya G. S., Prachiti P. K.

**Affiliations:** 1 National Centre for Earth Science Studies, Trivandrum, 695011 India; 2 CSIR-National Geophysical Research Institute, Hyderabad, 500 007 India; 3 Present Address: GITAM University, Nagadenehalli, Bangalore rural, Bangalore 561203, India

**Keywords:** Solid Earth sciences, Geodynamics, Tectonics

## Abstract

The Western Ghats is one of the largest escarpments on earth, containing Reunion plume derived Deccan Traps, it is an excellent example to probe epeirogenic uplift, extension and subsidence in volcanic continental margins. The most continuous unbiased stratigraphic section of basalt down to the basement within a 1250 m drill hole of the Continental Scientific Deep Drilling Project is a valuable resource to investigate the above aspects. The flows across the entire drill core are geologically subaerial in character with basement exposed ~300 m below the mean sea level; they clearly display more evolved compositions from primary melts of mantle in terms of petrology, and only a single geomagnetic polarity transition in palaeomagnetic data. These results, combined with existing geological and geophysical data, constitute a multi-method approach that demonstrates (a) igneous underplating caused uplift prior to frequently suggested flexural isostasy (b) plume impact and eruption are near-simultaneous and extension/rifting essentially followed soon after volcanism and (c) lithosphere beneath the continental margin, while returning to normal temperatures following the Seychelles-India breakup, experienced thermal collapse and subsidence causing slumping of basalt basement below sea level.

## Introduction

**Figure 1:**
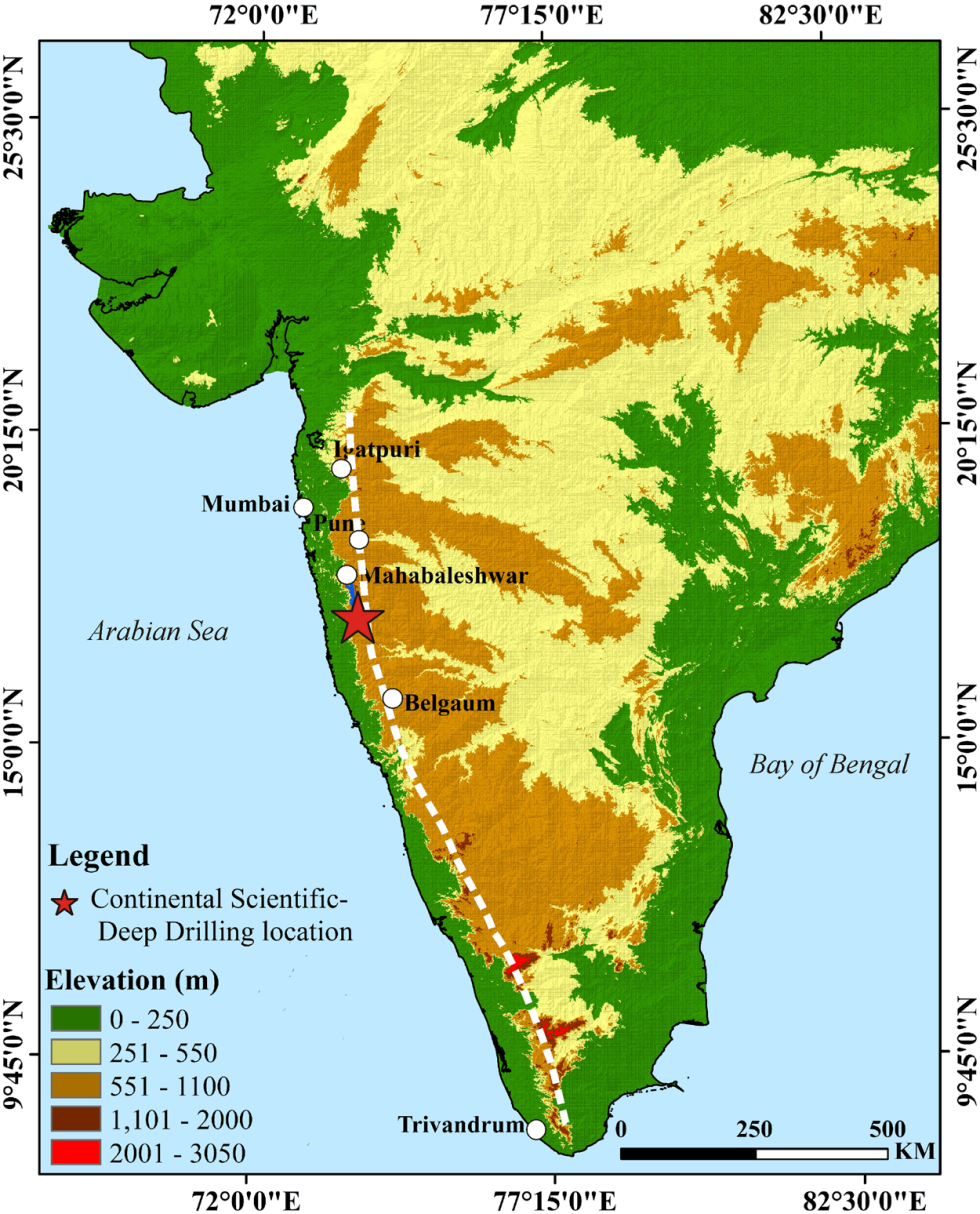
Topographic map of India based on Shuttle Radar Topography Mission (SRTM) data (https://earthexplorer.usgs.gov) using the 3D Analyst tool in ESRI-ArcGIS Version 10.3 Software. The Western Ghats escarpment is demarcated as a dashed white line.

The Western Ghat (WG) escarpment was developed from rifting and separation of India from the Seychelles and is closely linked with the late Cretaceous (~65–66 Ma) Deccan flood basalt eruption; it demarcates the low-lying seaward coastal plains in the west from the elevated plateau to the east (Fig. 1). It is one of the largest escarpments on a volcanic continental margin in the world with over 1500 km strike length paralleling the coast and elevations ranging beyond 1 km. Therefore, this is a classic example to investigate the relationship between volcanism, plateau uplift and extension (and rifting) on volcanic continental margins. Many investigations pertain to sustained erosion and retreat of the escarpment much to the east^1–3^. These studies attribute the uplift to flexural isostasy as a result of onshore denudational unloading and offshore loading in the sedimentary basins; a few others invoked neotectonic activity for the uplift^4,5^. However, several lines of evidence indicate that flexural loading alone cannot be responsible for the regional uplift (~3 km onshore unroofing is required to account for sediment loading)^6,7^ and it requires a preexisting elevated rift flank in addition to flexural uplift. Furthermore, the uplift and elevation in a non-volcanic rift environment is not commensurate with that found in the WG and such uplift can be linked to the development of a volcanic rift margin^8^. McKenzie^9^ was the first to revive a century old idea that igneous underplating is a possible mechanism for the epeirogenic uplift in regions other than plate boundaries. That is, the rift flank topography on the west coast may have close link with the Deccan flood volcanism.

There is also considerable debate on the extension/rifting and volcanism chronology. One view is that rift-margin igneous provinces form by decompression melting of a hot plume head beneath a region already undergoing extension^10^. Alternatively, the plume impact model^11^ proposes that large-scale volcanism results from melting of upwelling asthenosphere, leading to thinning of lithosphere and thereby extension and rifting. In either case, magmatic underplating is valid and may have induced rift flank uplift. Although a few isolated studies indicate magmatic underplating on the western margin of India, its linkage to uplift remained elusive. For example, a recent study of receiver function analysis^12^ suggests underplating in the Kutch region, but the region is much (>150 km) to the west of the escarpment in the north and it also experienced multiple tectonic episodes that may relate to different stages of the Gondwana breakup since the Triassic. The recent Continental Scientific Deep Drilling at Koyna (Fig. 1) within the WG^13^ gives a unique opportunity to access every successive flow of the stratigraphic section of the Deccan eruption down to the basement. In addition, geophysical investigations to bring out crustal structure in the Indian peninsular cover the WG^14–18^. Here, we assess the magmatic underplating model^9^ for explaining the rift-flank uplift and its mechanism along the volcanic continental margin using new and simple geological, petrological and palaeomagnetic data of the Deccan basalt down to the basement along the longest, continuous section of a drill core (1250 m; KBH-7) and by coupling the interpretations with the available geochronological, sedimentalogical, seismological and gravity results across the WG region. The WG is an ideal location for such a study using multi-method approach because a wide range of datasets are available.

## Results and Description of Samples of the Scientific Deep Drilling

The KBH-7 core was drilled at an elevation of 949 m above mean sea level (MSL).The basalt sequence along the core is demarcated into 37 flows although this number varies between different authors. Individual flows (5–85 m thick), usually demarcated by red boles, generally are massive at the bottom and vesicular and/or amygdaloidal at the top. The KBH-7 core encountered a basalt-granite gneiss interface at 300 m below the present day MSL; eight other drill holes around KBH-7 also encountered basement at closely comparable depths (~250–350 m below MSL). Despite this, the drilled basalt is subaerial and does not display subaqueous signatures like pillow structures or spilitisation. In contrast, the final phase of trachyte-rhyolite-basalt volcanism in the Bombay region recorded spilitisation^19^. There is no stratigraphic discontinuity, like in other parts of the WG, which indicates an erosional feature and discounts a major tectonic or structural control for the formation of the escarpment. Elsewhere, normal listric faulting is common roughly parallel to the coast in the Konkan region, and the traps are downthrown to the west along the N-S normal faults^20^ suggesting an east-west extension. The KBH-7 did not encounter dyke intrusions.

**Figure 2:**
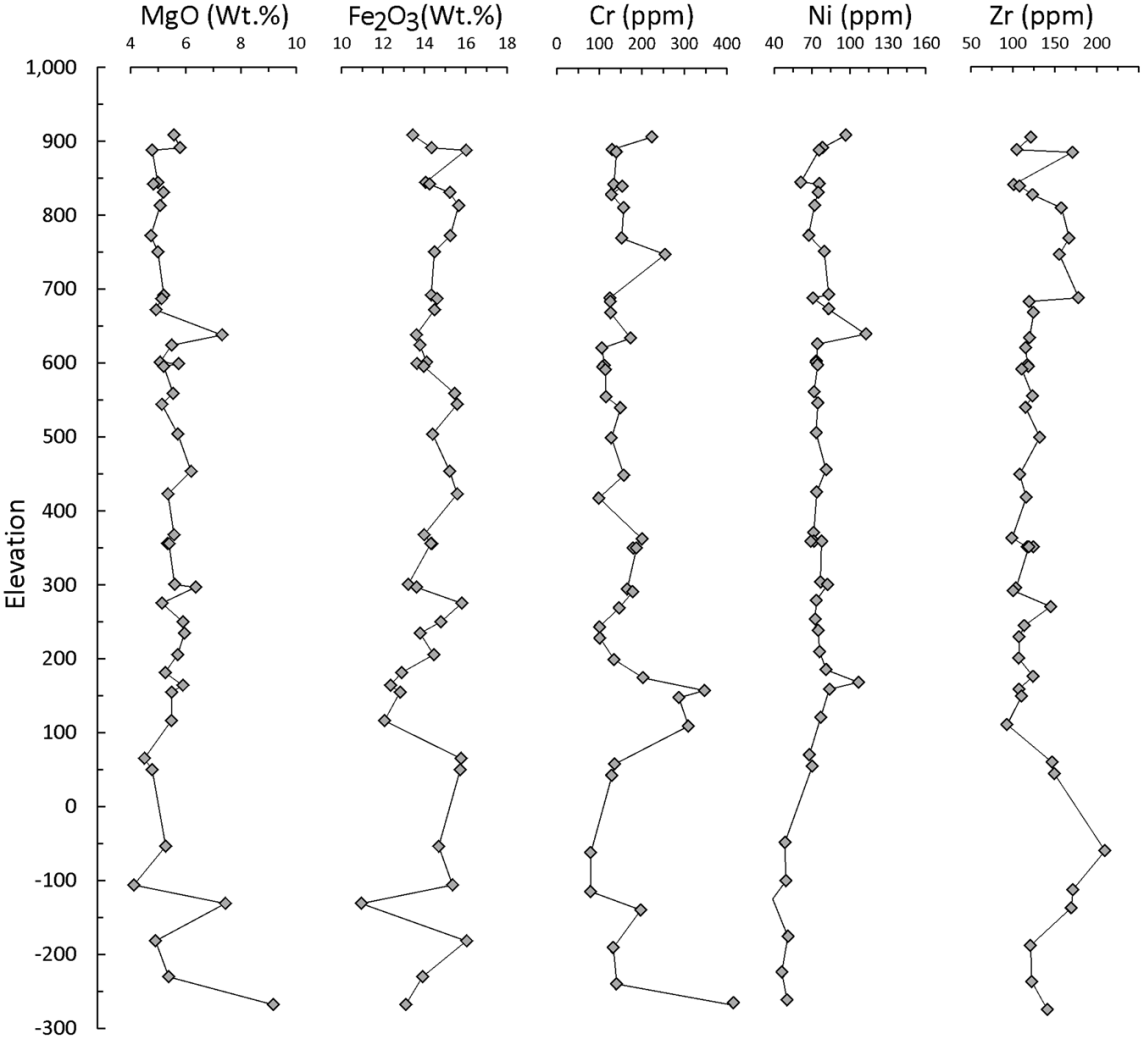
Variation of critical elemental concentrations across the drill hole KBH-7 from the surface down to the basement. The elevation (in meters) on the Y axis is with respect to MSL.

**Figure 3:**
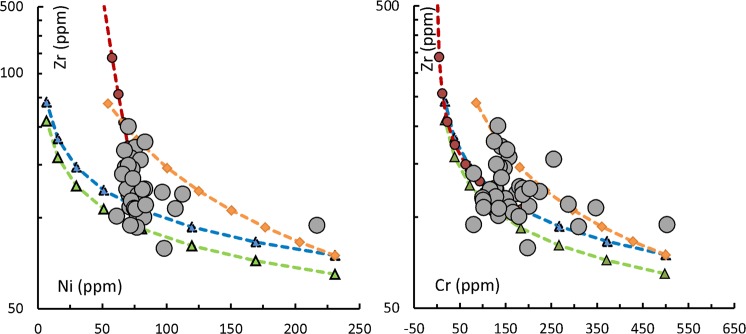
Zr vs Ni (left) and Cr (right) variations of KBH-7 drill core samples along with Batch melting model partial melting curves and Rayleigh fractionation modelled trends. The tie lines connecting Blue and green triangles denote picrite (ol:cpx:pl = 25:35:40) fractionation at 10% increments from 10% and 12% partial melts respectively of peridotite mantle (Zr: 8.42 ppm; Cr: 2645 ppm and Ni: 1985 ppm; values from Lyubetskaya and Korenaga^47^). The tie lines connecting the closed circles denote gabbro (ol:cpx:pl = 2:40:58) fractionation after 30% picrite fractionation. Tie lines connecting closed diamonds denote picrite (ol:cpx:pl = 25:35:40) fractionation (at 10% interval) from 10% partial melts. Partition coefficients for partial melting and fractionation modeling are taken from https://earthref.org/KDD/. It is seen that the KBH-7 Deccan samples are the variants between picrite-gabbro fractionated partial melts and direct picrite (with different proportions of mineral phases) fractionated partial melts.

Selected elements from geochemical data (determined by XRF and ICPMS methods) on 45 samples, taking at least one sample from each flow within the KBH-7 hole, are plotted in Fig. 2. The samples are very monotonous in composition and are Fe-rich sub-alkaline tholeiitic basalts; intermediate, silicic or alkali compositions are totally absent. The samples are low in MgO (<7.31 wt %). Correspondingly, their Mg numbers (Mg/(Mg + Fe^+2^) are also lower (0.52-0.36 with an exception of two samples showing values of 0.60 and 0.62) compared to the values (≥0.68) of primitive melts of the mantle. Both Cr and Ni generally behave similarly in basaltic melts and decrease along the liquid line of descent with fractionation of ferromagnesian mineral phases. Abundances of both these elements are also low in the entire KBH-7 basalt section (Cr < 300 ppm except in two samples and Ni < 112 ppm) compared to the primary melt compositions (Cr ~450–500 ppm and Ni ~250 ppm); these chemical signatures are in agreement with olivine rich (picrite) fractionation from primitive mantle melts (Fig. 3).

**Figure 4:**
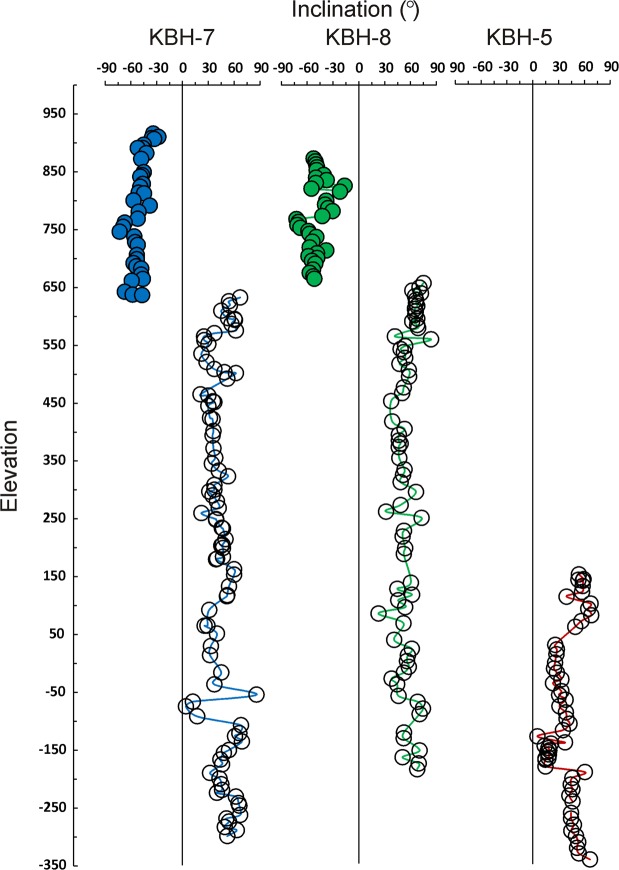
Characteristic magnetization inclinations of Deccan basalt down to basement interface along three drill holes (KBH-7, KBH-8 and KBH-5A) against the mean sea level elevation. It is seen that only one geomagnetic polarity transition recorded between normal (closed symbol) and reverse (open symbols) polarity at ~640–650 m above MSL. The drill hole (KBH-5A) drilled at an elevation of 152 m MSL did not record the top normal polarity because the polarity transition is at ~640–650 m MSL elevation.

Characteristic magnetisations were determined from azimuthally unoriented samples of each flow across KBH-7 and two other drill cores (KBH-5A and KBH-8) through detailed alternating field demagnetisations at close intervals (2.5 mT) until the stable magnetisation is achieved (details are in a manuscript under preparation). The inclination data are used here for determining the geomagnetic polarity and are plotted against MSL elevation in Fig. 4. The agreement between the three drill holes is very remarkable. The flow samples from surface down to ~640–650 m MSL show normal polarity with upward inclination (I = −50 ± 10°; n = 36) and thereafter the flow samples down to the basement show a reverse polarity with downward inclinations (I = +44 ± 14°; n = 90). These results establish unequivocally a single geomagnetic polarity transition during the eruption of the entire section of the basalt. A recent study based on mafic dykes near Bombay^21^ argues for four polarity transitions, but the interpretations are in conflict with the stratigraphic observations and also lack radiometric age support. The latest high-precision age data^22–24^ place the peak Deccan eruptions at about 66 Ma although a few ^40^Ar/^39^Ar ages range up to 68 Ma (see Fig. 2)^25^. In a comparison with geomagnetic polarity time scales, many authors^26–28^ argue that the eruptions represent 29N and 29R while some others relate them to older polarities of 30N/R or 31N/R^21^ (also see reference 28 for more details). In either case, the polarity data suggest that the whole eruption occurred within a short duration (<1 Ma considering 29N and 29R or <3 Ma; considering 31N and 31R). A short lower normal polarity transition is reported only from complex tectonic and structural regions.

### Uplifted rift-flank topography and underplating

Mantle plumes produce massive quantities of partial melt and a part of it penetrates to the surface to produce flood basalts^10,29^. Although estimates of volume of melt produced is not known, the entire volume of surface eruptions of the Deccan cannot account for the volume of magmas that can be generated by hot rising mantle plume. The unequivocal evidence for the evolved nature through picrite fractionation of the KBH-7 samples of the Deccan traps and similar compositions of surface samples^30^ and related igneous occurrences further south along the coast^31^, clearly indicate formation of picrite at depth in the continental crust prior to surface eruption. Such picrite, whose density is greater than the upper crust could form a layer between the crust and mantle^9^. A model based on petrological grounds^29^ also argued for magma differentiation and the formation of a picrite layer above the Moho for the Lisotho and Parana lavas and the Rooi Rand dolerites. Receiver function analysis of the teleseismic data and tomographic imaging^17,18,32^ and the Deep Seismic Sounding (DSS) profiling^14^ predicts the presence of such a horizontal layer at the crustmantle interface under the WG, with the Moho depth off-sets ranging up to 8 km. Gravity modeling coupled with inferred seismic velocity structure indicates the presence of a high density material (3.1 g/cm^3^) at the crust-mantle interface^15^. Because there is (i) no difficulty to produce uplift by magmatic intrusion at the crust-mantle interface, (ii) evidence for differentiation of olivine rich picritic magma before the magma reaching the surface and (iii) extensively widespread surface volcanism in the form of Deccan flood basalt, it is not difficult to propose uplift as a result of magma intrusion. Mantle heterogeneities modeled using viscous flow predicted dynamic surface topography of strong surface uplift along the western coastal margin of India coinciding with the timing of the Deccan eruption^33^. Magmatic underplating is cited to explain uplift as a result of the 260 Ma Emeishan lava eruptions in western margin of the Yangtze Craton, southwest China^34^. The Deccan is among the two largest flood volcanic eruptions on Earth in the last 200 Ma and the removal of melt naturally depletes the upper mantle and hence reduces its density. Presence of such a low density mantle layer (3.2 g/cm^3^) is inferred in the upper mantle beneath the WG region based on gravity data^15^. This depleted mantle also can cause uplift as can the crustal thickening by the addition of igneous rock to the crust^10^. Thus, the conclusion from this work presents a strong case suggesting large igneous intrusions are a major cause for epeirogenic uplift, particularly when flood basalt magmatism is present in the regions of uplift.

Flexural isostatic compensation in response to early Cenozoic erosion and deposition is evident by the drastically high rate of clastic sediment loading in the offshore basins as a result of high rate of rift-flank denudation in the WG during the Paleocene (66-56 Ma^6,7^). Modeled thermal histories of the apatite fission track dates also suggest higher rates of denudation at the start of the Cenozoic^35^. These studies also emphasise significant Neogene uplift in the WG escarpment. Mass balance studies and numerical modeling of flexural responses to onshore denudational unloading and offshore sediment loading infer that flexural isostasy alone is not adequate to explain the quantum of offshore sediment deposition^6,7^ and requires a pre-existing elevated rift flank. We propose here such elevated rift flank topography was formed by igneous underplating at the crust mantle interface and development of a low density depleted mantle layer on removal of melts at the time of up-rise of mantle and flood basalt eruption. An argument against such a mechanism prefers lithospheric necking for the uplift in the WG escarpment^36,37^. This argument assumed (i) the total uplift is due to underplating, overlooking the well recorded Neogene uplift^6,7^ and (ii) the uplift of rift flank topography was the result of the c. 88 Ma Madagascar-India breakup, contrary to the c. 65 Ma breakup between the Seychelles and India as suggested by many works^10,30,31,38^. Our present work provides clear evidence for igneous underplating based on simple petrological and geological grounds, and is supported by other published geological and geophysical results^12,14–18,29,30^.

### Volcanism vs extension chronology

The results of present study also have implications for the volcanism and extension chronology in uplifted plateau regions on the volcanic continental margins. Notably, no silcic and alkaline magmatism is found across KBH-7 and the eruption of Deccan basalt is rather rapid as recorded by only a single geomagnetic polarity transition across the drill holes down to the basement; furthermore, the feeder dykes of the Deccan flows are randomly oriented^39^. These factors indicate that there was insufficient time for plume incubation and/or extension prior to volcanism. Intense extension and rifting followed soon after the initiation of adiabatic mantle upwelling and melting is well recorded by the preferred orientation of silicic to alkaline dykes cross-cutting the Deccan flows, slightly younger ages (~64-62 Ma) of the silicic/alkaline eruptions/intrusions in the Deccan province^40,41^, spilitisation in the final phase of volcanism as a result of rapid subsidence or eruption after westward down through of basalt along normal faults developed by rifting in the Bombay region, and a lack of prominent seaward dipping reflectors off the Indian west coast^42^. A similar chronological sequence is evident in the case of the Kerguelen plume activity also in the eastern part of India. ^40^Ar/^39^Ar ages of the igneous activity in eastern India show younger 114-105 Ma ages for alkaline magmatism compared to the slightly older 118-115 Ma peak tholeiitic flood basalt eruption^43^. Significant extension and crustal thinning followed, rather than preceded, the eruption of the typical continental flood basalt of the Columbia River volcanic province^39,44^. These interpretations are in agreement with the findings that continental uplift has often been reported prior to rifting^45^.

### Thermal collapse and slumping down of basalt-basement interface

Finally, it is pertinent to offer an explanation for the present location of basalt-basement interface much below the MSL despite subaerial characteristics all along the Deccan section down to the basement. The heat flux associated with magma underplating decays very slowly on a time scale of the order of 50 to 100 Ma, but the heat due to upwelling of hot asthenosphere decays very fast^10^, particularly in case of the Reunion plume which moved away from its initial rising location. The lithosphere in western India, upon gradual cooling due to igneous underplating, became denser and the shallow basins above steadily subsided and progressively filled with shallow-water sediment derived from onshore denudation during the Palaeocene. As the temperatures returned to normal, the lithosphere experienced thermal collapse and subsidence, and thereby slumped down the basalt-basement interface far below to the level observed today in the drill cores (250–300 m below MSL). This change is also marked by the cessation of clastic sedimentary deposition in the offshore basins^7^. Although sea level changes could be argued as a possibility for the basalt-basement contact below MSL, the higher sea levels in the late Cretaceous (at least 200 m above the present day MSL^46^) do not favour the argument. Flexural isostasy appears to have become an important factor, and may have overtaken the influence of thermal subsidence, if any, by the Neogene period. The uplift appears to have resumed, as reflected by a second phase of clastic sediment deposition^4,6,7^, and it could be responsible for the neotectonic activity. The on-going micro-seismic activity and erosion/denudation in the WG indicate that the epeirogenic movements of flexural isostasy are still active in the Western Ghats and the denudation/erosion is likely driven mainly by uplift rather than climate.

## Conclusion

This paper presents new petrological, geological and palaeomagnetic data from samples of the Deccan basalt within a continuous drill core section (s) of the Continental Scientific Deep Drilling Project in the Western Ghats, India. The data are combined with existing geological and geophysical results to explain the processes, chronological order and relationships between volcanism, uplift and extension/rifting along this volcanic continental margin. The basaltic drill core section is entirely subaerial in character and its interface with the basement is much below the mean sea level (~300 m); the section is petrologically more evolved from primary mantle melts and records only a single geomagnetic polarity transition. The main interpretations are that (a) igneous underplating was a cause of initial epeirogenic uplift in the WG, (b) extension/rifting followed shortly after volcanism and (c) thermal collapse and subsidence caused slumping down of basalt basement below sea level, while returning to normal temperatures after the Seychelles-India breakup.
